# Performance Differences Between Spanish AzBio and Latin American HINT: Implications for Test Selection

**DOI:** 10.3390/audiolres15050129

**Published:** 2025-10-02

**Authors:** Chrisanda Marie Sanchez, Jennifer Coto, Sandra Velandia, Ivette Cejas, Meredith A. Holcomb

**Affiliations:** 1 Department of Otolaryngology, University of Miami, Miami, FL 33136, USA; jennifercoto@med.miami.edu (J.C.); svelandia@med.miami.edu (S.V.); icejas@med.miami.edu (I.C.); meredith.holcomb@med.miami.edu (M.A.H.); 2 Department of Pediatrics, University of Miami, Miami, FL 33136, USA

**Keywords:** Spanish, audiology, Spanish AzBio, Latin American HINT, healthcare disparities, Hispanic/Latino, cochlear implant candidacy

## Abstract

**Background/Objectives:** Spanish-speaking patients face persistent barriers in accessing equitable audiological care, particularly when standardized language-appropriate tools are lacking. Two Spanish-language sentence recognition tests, the Spanish AzBio Sentence (SAzB) and the Latin American Hearing in Noise Test (LAH), are commonly used to evaluate speech perception in adults with hearing loss. However, performance differences between these measures may influence referral decisions for hearing intervention, such as cochlear implantation. This study compared test performance under varying noise and spatial conditions to guide appropriate test selection and reduce the risk of misclassification that may contribute to healthcare disparities. **Methods:** Twenty-one bilingual Spanish/English speaking adults with normal bilateral hearing completed speech perception testing using both the SAzB and LAH. Testing was conducted under two spatial configurations: (1) speech and noise presented from the front (0° azimuth) and (2) speech to the simulated poorer ear and noise to the better ear (90°/270° azimuth). Conditions included quiet and three signal-to-noise ratios (+10, +5, and 0 dB). Analyses included paired *t*-tests and one-way ANOVAs. **Results:** Participants scored significantly higher on the LAH than on the SAzB across all SNR conditions and configurations, with ceiling effects observed for the LAH. SAzB scores varied by language dominance, while LAH scores did not. No other differences were observed based on any further demographic information. **Conclusions:** The SAzB provides a more challenging and informative assessment of speech perception in noise. Relying on easier tests like the LAH may obscure real-world difficulties and delay appropriate referrals for hearing loss intervention, including cochlear implant evaluation. Selecting the most appropriate test is critical to avoiding under-referral and ensuring Spanish-speaking patients receive equitable and accurate care.

## 1. Introduction

Hearing loss is one of the most prevalent sensory disabilities, significantly impacting communication, social integration, and overall quality of life. The World Health Organization (WHO) projects that by 2050, nearly 2.5 billion people will have some degree of hearing loss, with at least 700 million requiring hearing rehabilitation [1]. In the United States (U.S.), nearly one in seven Hispanic/Latino adults experience hearing loss, a rate comparable to the non-Hispanic population [2]. Furthermore, the need for accessible audiological care is becoming increasingly urgent as the Hispanic/Latino population in the U.S. continues to grow.

Currently, 62.6 million individuals in the U.S. identify as Hispanic, comprising 18.9% of the total population [3]. The number of individuals aged five years and older who speak Spanish at home has grown 275.6% between 1980 and 2019 [3]. Despite this demographic shift, audiological services have not evolved to adequately meet the linguistic and cultural needs of Spanish-speaking patients. Specifically, there is a notable lack of standardized Spanish-language speech perception measures, which are essential for evaluating hearing loss and guiding treatment decisions [4,5].

For sentence recognition, Rivas and colleagues developed the Spanish AzBio Sentence (SAzB) Corpus which were deemed equivalent in terms of difficulty and considered to be of benefit when assessing native Spanish speakers in both research and clinical settings [6]. The SAzB contains sentences of varying length (7–12 words) with natural intonation, multiple syntactic structures, and a mix of high- and low-frequency vocabulary, reflecting conversational, everyday Spanish. It is recorded by four native speakers (two male, two female), which increases talker variability and better approximates real-world listening. Sentences often incorporate descriptive language and multiple content words. For example, a SAzB sentence might read “Ayer por la tarde fuimos al mercado a comprar verduras frescas” (“Yesterday afternoon we went to the market to buy fresh vegetables”), which combines a time-related introductory clause with descriptive vocabulary and natural prosody.

Prior to the availability of this test, the Latin American Hearing in Noise Test (LAH) [7] was primarily utilized to assess speech recognition for Spanish speakers. The LAH consists of shorter sentences (5–9 words) with simpler grammatical structures and more predictable, high-frequency vocabulary. All sentences are spoken by a single monotone male talker at a controlled pace, with speech-shaped noise as the masker. For example, an LAH sentence might read “El niño juega con su pelota” (“The boy plays with his ball”), a syntactically simple subject–verb–object construction with minimal phonetic variability.

The SAzB and LAH differ in complexity of sentence structure, length of sentences and variability in speakers (LAH with a monotone male voice and SAzB with four different speakers—two male and female). These linguistic and acoustic differences, including sentence length, syntactic complexity, vocabulary diversity, number and variability of talkers, and type of background noise, can influence perceptual difficulty, cognitive load, and ultimately test performance.

Based on existing studies, patients will likely score better with the easier task, LAH, than the more difficult task, SAzB, which could negatively impact access to appropriate treatment options for hearing loss. A 2023 survey of US CI audiologists concluded that while many clinicians utilize both SAzB and LAH interchangeably, there remains a lack of notable research on which test measure to utilize clinically and which protocol to follow as best practice [5]. More recently, Velandia and colleagues (2024) showed ceiling effects for LAH and cautioned against use of this test measure for CI candidacy; however, they did not include SAzB in their study as it was not available at the time of data collection [8]. While there is a paucity of research on the comparison of these test measures and when to utilize them, it is well known that the English version of AzBio [9] is recommended over the English HINT [10], due to known ceiling effects and lack of real-world comparison for HINT [11,12].

Regarding bilingual listeners, Rodriguez and Shader compared performance on the AzBio tests (English and Spanish versions) with individuals to assess the effects of language dominance on testing outcomes. Their findings suggested that bilingual listeners scored comparably on the English and Spanish versions of the test. However, individuals with Colombian dialect scored the highest on the SAzB and the lowest on the English version, suggesting that dialect plays an integral role in these testing outcomes. As a result, dialect considerations may affect test selection, and clinicians should consider these factors when determining the ideal test battery for Spanish-speaking and bilingual patients [13].

Furthermore, speech testing protocols are often adapted to accommodate the specific needs of individuals with different types of hearing loss. For patients with bilateral hearing loss, speech testing is typically conducted with the signal presented directly in front of the patient. However, for individuals with unilateral hearing loss, such as single-sided deafness (SSD) or limited usable hearing unilaterally (LUHU), the speaker configuration may vary to better assess functional hearing. Despite the increasing use of Spanish-language speech perception measures like the SAzB and LAH, no studies to date have documented performance differences across these tests or speaker configurations in Spanish-speaking patients. This gap in the literature underscores the need for research that evaluates how spatial configuration and test selection impact speech perception outcomes in this growing population.

The purpose of this study was to compare the overall performance of Spanish speaking individuals when tested with the SAzB versus the LAH when speech and noise is both collocated as well as spatially separated.

## 2. Methods

### 2.1. Participants

Twenty-one adult participants who self-identified as fluent Spanish speakers were prospectively recruited and consented for this study. Inclusion criteria required participants to have normal bilateral hearing sensitivity, confirmed via audiometric evaluation, to ensure symmetric baseline auditory function before testing. Normal-hearing participants were selected to establish normative reference data for these Spanish-language speech perception measures without the variability introduced by differing degrees or configurations of hearing loss. These normative data can serve as a baseline for interpreting performance in future studies involving clinical populations. Participants completed a demographic survey via RedCap. Information on sex, race, ethnicity, country of birth, parental country of birth, primary language spoken, first and second language proficiency, subjective language dominance, level of education, and occupation was collected. This study was approved by the University of Miami Institutional Review Board (protocol #20230168).

### 2.2. Speech Perception Testing Conditions

Speech perception testing was completed in a calibrated sound-treated booth using two clinically available Spanish-language recorded speech materials: the SAzB and the LAH. Testing was presented at 60 dB SPL under two listening conditions in the free field, bilateral normal hearing condition and simulated unilateral hearing loss condition.

- Bilateral Normal Hearing Condition—Participants were tested with both ears unoccluded and unaided.
- Simulated Unilateral Hearing Loss Condition—A deeply inserted earplug combined with a noise-reducing earmuff was placed on the right ear to simulate unilateral hearing loss, disrupting binaural cues. Of note, this plug and muff configuration resulted in a unilateral, flat moderate hearing loss (mean of 52.5 dB pure tone average; 500–4000 Hz). Monaural testing with contralateral masking was not feasible in this study’s spatially separated speech-in-noise condition because the speech and noise stimuli were already presented through both available audiometer channels. Adding masking would have required a third channel, which was not available on the clinical audiometers used for this study. Therefore, a plug and muff configuration was selected as a practical and clinically accessible method to reduce input to the poorer ear while preserving the intended spatial separation of speech and noise. While this simulation cannot replicate the long-term perceptual and cognitive effects of actual hearing loss, it provides a controlled means of assessing how spatial separation and test type influence performance when binaural hearing is disrupted.

### 2.3. Test Configurations

**Figure 1:**
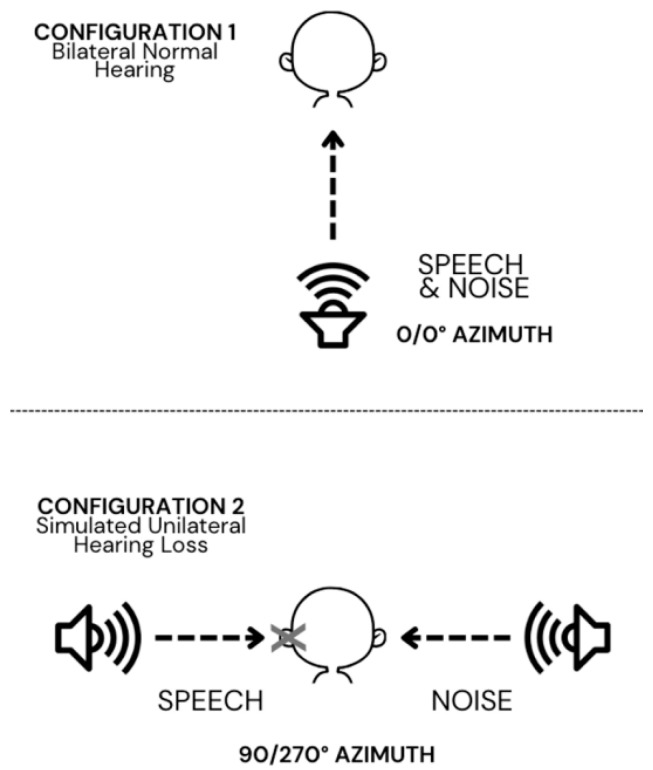
This figure describes the two testing configurations. Configuration 1 represents the normal hearing condition with speech and noise coming from the front speaker (0/0° azimuth). Configuration 2 represents the simulated hearing loss condition with speech and noise spatially separated (90°/270° azimuth).

Speech perception testing was performed across two different spatial configurations and four signal-to-noise ratio (SNR) conditions per configuration (Figure 1). Each participant was tested with the SAzB and LAH in two spatial configurations: (1) 0° azimuth (speech and noise collocated to the front) and (2) 90°/270° azimuth (speech and noise spatially separated). These tests were performed in quiet, +10 dB SNR, +5 dB SNR, and 0 dB SNR. This entire protocol was performed in a bilateral normal hearing condition and then repeated in a simulated hearing loss condition (see Section 2.2). Participants’ speech perception scores (percent correct) were recorded for each test condition and configuration to assess the impact of speech perception in noise as it related to spatial hearing and simulated unilateral hearing loss on Spanish-language speech perception.

## 3. Results

Twenty-one adults with a mean age of 34.29 years (*SD* = 8.98) were enrolled in the study (*n* = 17; 81% female). Most participants identified as Hispanic (*n* = 19; 90.5%) and white (*n* = 21; 100%), were born in the U.S. (*n* = 13; 61.9%) and held an Associate’s Degree or higher (*n* = 17; 81%). Maternal birth country was endorsed as predominantly from other countries (*n* = 19), including Colombia (*n* = 5), Nicaragua (*n* = 5), and Cuba (*n* = 3). Spanish was the language most commonly endorsed as their first language spoken (*n* = 17; 81%). However, most participants reported English as their current preferred language (*n* = 14; 67%), 28.6% as Spanish (*n* = 6), and 4.8% as other (*n* = 1). In terms of language dominance, over half rated themselves as English dominant and secondary Spanish (*n* = 11; 52.4%). See Table 1 for further demographics.

Paired sample *t*-tests revealed differences between the SAzB and LAH with all participants scoring significantly higher on the LAH at every SNR condition, compared to the SAzB (see Table 2). On average, participants scored 2.85–28.25 points higher on the LAH compared to the SAzB. All participants achieved 100% on the LAH at most SNR conditions except at 90 + 5 (*M* = 99.85) and 90 + 0 (*M* = 91.84). Next, multiple One-Way ANOVAs were conducted to examine SAzB scores by language dominance. Of note, SAzB scores by language dominance could not be analyzed at SAzB0 + quiet and SAzB90 + quiet as all participants scored at the ceiling. ANOVA results revealed that those reporting Spanish dominance and English secondary had the highest scores, followed by equal language dominance, and English dominant and Spanish secondary (see Table 3 for further details). On the contrary, there were no statistically significant differences between LAH scores according to language dominance (see Table 4). Means between the groups ranged from 0–12.2. Interestingly, only three SNR conditions (LAH + 0, LAH90 + 5, and LAH90 + 0) were included in the analyses examining the LAH by language dominance as all participants scored at the ceiling for the remaining SNR conditions. Additionally, when examining SAzB and LAH scores by highest education, One-Way ANOVAs revealed no statistically significant differences (*p* = 0.12–0.89).

## 4. Discussion

This study provides new insights into normative performance on two commonly used Spanish-language sentence perception tests under varying noise conditions. Our results confirm that the SAzB test is more difficult than the LAH, particularly in challenging listening environments with more challenging SNRs. Participants reached ceiling effects in the normal hearing condition and unilateral HL condition for both SAzB and LAH in quiet. This aligns with previous reports for the English versions of AzBio and HINT sentence test measures [14,15]. Ceiling effects were also observed in our study for the LAH in noise conditions of 0° azimuth at +10 and +5 SNR, and the spatially separated 90/270° azimuth condition at +10 SNR. Velandia and colleagues (2024) reported similar LAH results in 24 Spanish-speaking adults with a cochlear implant who were tested in quiet and +5 SNR at 0° azimuth. To our knowledge, this is the first report in the literature showing ceiling effects for LAH in the test condition of +10 SNR spatially separated 90/270.

It is likely that the differences noted for SAzB and LAH ceiling effects are due to LAH having a slower rate of speech, single speaker configuration, easier vocabulary, simple grammatical structure, and less words per sentence when compared to the SAzB. In addition, the background noise used for LAH is speech-shaped noise rather than multi-talker babble used for SAzB. This makes the speech-in-noise condition inherently easier for listeners, as they do not have to separate target speech from competing speech. Given these observations, we agree with Velandia and colleagues that clinicians should be cautious about utilizing the LAH as a standard default test, as it may inflate performance and underrepresent patient difficulty [8]. This could negatively impact access to hearing loss treatment options like cochlear implantation as LAH scores may limit candidacy qualification. Therefore, SAzB is the preferred sentence test in the pre- and post-CI test battery for the Spanish speaking population. Additionally, given the high rate of ceiling performance with the LAH across all noise conditions, this test measure should primarily be used as a reference for monitoring progress and establishing a baseline for speech perception abilities in those patients who have significant difficulty with the SAzB, rather than establishing candidacy.

The decision to include only normal-hearing participants was intentional to first establish normative performance benchmarks without the confounding effects of varied degrees, configurations, or durations of hearing loss. These baseline data provide a controlled reference point against which future clinical populations, such as hearing aid or cochlear implant users, can be meaningfully compared.

Understanding which test to administer first and at which SNR patient performance begins to degrade is critical for effective clinical decision-making. This study uniquely contributes to the field by establishing performance benchmarks for normal-hearing Spanish-speaking adults. In addition, the simulated unilateral hearing loss condition results allow for clinical guidance examining how speech perception, particularly in noise, is affected by disrupted binaural cues, as would be the case for unilateral listeners. Prior studies, such as Sanchez et al. (2023) and Turnbull et al. (2023) have primarily relied on survey data to assess clinical practices for Spanish-speaking adults and children and base recommendations on those findings [4,5]. Access to normative data enables clinicians to set realistic expectations for Spanish-speaking patients and tailor rehabilitative strategies accordingly.

Regarding hearing intervention that involves cochlear implant candidacy, the landscape continues to evolve alongside the increasing diversity of the U.S. population. Even so, there remains a significant need for standardized guidelines and assessment tools for evaluating Spanish-speaking cochlear implant candidates, as this candidacy is often based off speech perception. Current recommendations include utilizing a 3-frequency pure tone average of 60 dB HL or worse [16,17] regardless of unaided speech scores to identify potential cochlear implant candidates. Aided bisyllabic Spanish word scores of 50% or worse have been suggested in the literature [18,19,20] as the metric for candidacy determination. Even with current recommendations emphasizing the importance of incorporating language and cultural considerations into clinical practice, there is limited guidance on how to effectively assess Spanish-speaking individuals audiological performance [21]. Given this gap, future studies should aim to establish a structured Spanish test battery that accounts for linguistic diversity while ensuring equitable access to cochlear implant candidacy evaluations, specifically for this population. Ensuring equitable assessment and treatment for Spanish-speaking patients is critical to mitigating existing healthcare disparities [3]. This study provides novel information regarding how to best assess speech perception for Spanish speaking adults in noise and will help provide consistency in clinical practice and improve patient outcomes across audiology clinics.

While this study used only adult participants, it should be noted that US audiologists utilize SAzB and LAH with older children and adolescents [4]. As such, incorporating our study’s recommendations should be considered for this population as well to ensure they receive appropriate testing and support for their auditory needs. Despite the availability of Spanish-language speech perception measures, many audiologists are not routinely administering these tests to Spanish-speaking patients [4,5]. This discrepancy suggests that beyond test availability, audiologists’ familiarity, training, and confidence in administering Spanish-language assessments may also contribute to healthcare disparities for the Spanish speaking population.

### Limitations

Despite its contributions, this study has several limitations. Simulated hearing loss was introduced via a deeply inserted earplug and muff, which served to disrupt binaural cues and present speech and noise in a spatially separated condition. As a result, this study does not fully replicate real-world unilateral sensorineural hearing loss SSD/LUHU or bilateral hearing loss conditions. We acknowledge that this short-term simulation does not account for the auditory deprivation, cortical reorganization, and cognitive adaptations that occur in individuals with longstanding hearing loss; thus, the results should be interpreted as representative of immediate perceptual effects rather than long-term functional outcomes. Despite this limitation, understanding the implications of these speech in noise measures and degradation of speech understanding on normal hearing individuals is a crucial first step in understanding baseline performance.

Additionally, while the SAzB test utilizes speech babble as a competing noise, the background talkers are speaking English rather than Spanish. Although this linguistic mismatch may not significantly affect outcomes, it does highlight a suboptimal test design that could influence real-world applicability. Future research should explore test modifications that better simulate real-world Spanish-speaking listening environments, including the use of multi-talker Spanish babble as a competing noise source.

The participant cohort of this study included bilingual Spanish/English speakers, which may reflect differences in performance when compared to monolingual Spanish speakers. SAzB scores varied according to self-reported language dominance, with those reporting Spanish dominance and English secondary performing highest, followed by participants with equal dominance, and those reporting English dominance with Spanish secondary. In contrast, language dominance did not significantly influence LAH scores. This difference may be attributable to the greater linguistic and acoustic complexity of the SAzB, which incorporates longer sentences, more varied grammatical structures, multiple talkers, and multi-talker babble. These factors may place higher demands on listeners’ language proficiency, as described in the Introduction. Additionally, subjects were only tested in Spanish, thus this study did not highlight comparative differences between English and Spanish performance with bilingual listeners. Including English-language sentence materials could have enabled a direct cross-linguistic comparison within this bilingual cohort; however, this was beyond the scope of the present study and remains an important avenue for future research. While studies have compared performance between English and Spanish testing on bilingual listeners [13], future studies should delve into the influence that dialect may play in outcomes for Spanish speakers.

Overall, this study highlights a need for a recommended test battery for Spanish speaking adults with hearing loss. While this study offers a significant step toward standardization, continued research is needed to refine testing measures, particularly for younger children, and to develop speech perception assessments that more closely reflect real-world listening environments. By addressing these ongoing challenges, future advancements can further enhance the effectiveness of clinical assessments for Spanish-speaking patients across diverse hearing healthcare settings.

## 5. Conclusions

The results from our study provide clinical guidance for a more equitable, systematic, and effective approach to speech perception testing for Spanish-speaking individuals, ultimately enhancing clinical outcomes and improving access to quality audiological care. Without widespread implementation and adherence to standardized protocols, Spanish-speaking patients may not receive equitable audiological care, potentially impacting clinical decision-making and intervention outcomes. Addressing this gap requires not only the development of structured testing guidelines but also increased education and training to ensure clinicians are equipped to effectively evaluate and manage Spanish-speaking patients.

## Figures and Tables

**Table 1:** Sample Demographic Characteristics.

Characteristic	*n* (%)
Mean Age (*SD*)	34.29 (*8.98*)
Sex Female Male	17 (81%) 4 (19%)
Highest level of education High school or GED Some college, no degree Associate’s degree Bachelor’s degree Master’s degree Doctoral degree	1 (4.8%) 3 (14.3%) 2 (9.5%) 5 (23.8%) 1 (4.8%) 9 (42.9%)
Ethnicity Non-Hispanic Hispanic	2 (9.5%) 19 (90.5%)
Race White	21 (100%)
Participant Birth Country USA Other Brazil Colombia Cuba Dominican Republic France Venezuela	13 (61.9%) 8 (38.1%) 1 3 1 1 1 1
Maternal Birth Country USA Other Brazil Colombia Cuba Dominican Republic El Salvador France Nicaragua Venezuela	2 (9.5%) 19 (90.5%) 1 5 3 1 1 1 5 1
First language spoken English Spanish Other	2 (9.5%) 17 (81%) 2 (9.5%)
Primary language spoken English Spanish Other	14 (66.7%) 6 (28.6%) 1 (4.8%)
Language Dominance English dominant, Spanish secondary Spanish dominant, English secondary Equal language dominance Other language dominant	11 (52.4%) 3 (14.3%) 6 (28.6%) 1 (4.8%)

**Table 2:** Paired sample *t*-tests between SAzB and LAH scores.

	Mean (*SD*)	*t*	*p*
SAzB0 + 10 LAH0 + 10	97.15 (4.69) 100.00 (0.00)	−2.72	0.01
SAzB0 + 5 LAH0 + 5	93.60 (6.17) 100.00 (0.00)	−4.64	0.001
SAzB0 + 0 LAH0 + 0	71.75 (13.11) 100.00 (0.00)	−9.64	0.001
SAzB90 + 10 LAH90 + 10	83.55 (14.15) 100.00 (0.00)	−5.20	0.001
SAzB90 + 5 LAH90 + 5	68.90 (17.05) 99.85 (0.67)	−8.19	0.001
SAzB90 + 0 LAH90 + 0	22.74 (11.56) 91.84 (10.81)	−27.16	0.001

**Table 3:** SAzB bio scores by language dominance.

	Spanish Dominant, English Secondary (*n* = 3) Mean (*SD*)	Equal Language Dominance (*n* = 6) Mean (*SD*)	English Dominant, Spanish Secondary (*n* = 10) Mean (*SD*)	*F*	*p*
SAzB0 + 5	99.33 (0.58)	97.17 (2.32)	90.2 (6.53)	5.63	0.01
SAzB0 + 0	82.67 (8.08)	79.17 (8.28)	65.5 (12.89)	4.34	0.03
SAzB90 + 10	95.33 (3.79)	94.0 (5.18)	74.3 (13.68)	8.34	0.01
SAzB90 + 5	84 (3.46)	80.50 (8.09)	57.10 (15.91)	8.78	0.01
SAzB90 + 0	31.67 (5.51)	25.67 (15.02)	15.50 (7.03)	3.79	0.05

*Note.* SAzB0 + quiet and SAzB90+ quiet could not be analyzed as all participants scored at the ceiling.

**Table 4:** LAH scores by language dominance.

	Spanish Dominant, English Secondary Mean (*SD*) (*n* = 3)	Equal Language Dominance Mean (*SD*) *(n* = 6)	English Dominant, Spanish Secondary Mean (*SD*) (*n* = 10)	*F*	*p*
LAH + 0	100.00 (0.00)	100.00 (0.00)	99.30 (1.25)	1.32	0.30
LAH90 + 5	100.00 (0.00)	100.00 (0.00)	99.7 (0.95)	0.42	0.66
LAH90 + 0	100.00 (0.00)	96.40 (4.62)	87.70 (12.95)	2.23	0.14

*Note.* LAH0 + quiet, LAH0 + 10, LAH0 + 5, LAH90 + quiet, and LAH90 + 10 quiet could not be analyzed as all participants scored at the ceiling.

## Data Availability

The data supporting the conclusions of this article will be made available by the authors on request.
